# Ocular Manifestations in Patients with Sensorineural Hearing Loss

**DOI:** 10.18502/jovr.v17i4.12321

**Published:** 2022-11-29

**Authors:** Haniah A. Zaheer, Deepika C Parameswarappa, Myra A. Zaheer, Jay Chhablani, Preeti Patil-Chhablani

**Affiliations:** ^1^Department of Ophthalmology, University of Pittsburgh School of Medicine, Pittsburgh, PA, USA; ^2^L.V. Prasad Eye Institute, Hyderabad, Telangana, India; ^3^School of Medicine and Health Sciences, George Washington University, Washington DC, USA; ^4^UPMC Eye Center, University of Pittsburgh, Pittsburgh, PA, USA

**Keywords:** Developmental Effects, Neuro-ophthalmology, Ocular Manifestations, Pediatric Ophthalmology, Sensorineural Hearing Loss

## Abstract

Identification of ocular manifestations in patients with sensorineural hearing loss (SNHL) can have a large impact on the outcome and treatment of pediatric patients. Due to the common co-incidence of ocular manifestations and SNHL in children, both ophthalmologic and hearing loss screening and routine examinations must be conducted to minimize adverse outcomes and worsening of pathology. Early evaluation and diagnosis is imperative for intervention and further development of the patient. Co-incidence requires a thorough evaluation that includes a comprehensive history, examination, and diagnostic testing. In this article, a literature review was conducted to analyze the presentations of various diseases and syndromes, such as Alport Syndrome, Waardenburg Syndrome, Norrie Disease, Usher Disease, Stickler Syndrome, Marfan Syndrome, Congenital Rubella, and Hereditary Optic Neuropathies. We divided the various ocular pathologies into anterior and posterior segment presentations and associated systemic findings for better understanding. Additionally, this review aims to include an update on the management of patients with both ocular and hearing loss manifestations.

##  INTRODUCTION

The co-incidence of ocular manifestations and sensorineural hearing loss (SNHL) in pediatric patients can have a large impact on the overall development of the effected child. Patients with SNHL largely depend on other senses, such as vision, to compensate for the loss of auditory input. The development of the brain relies largely on the senses of sight and hearing; therefore, it is imperative to ensure diagnoses that involve both hearing and visual impairment are identified during early childhood and appropriate interventions instituted to prevent further developmental delays in children.^[1,2,3,4]^ Previous studies have looked at the association of individual syndromes associated with both SNHL and ophthalmologic findings, as are described in this article [Table 1].^[5,6,7,8,9,10,11]^ However, to the best of our knowledge, there has yet to be a comprehensive review that discusses multiple syndromes with visual and SNHL findings, diagnostic and management options of these syndromes, and the implications on development for pediatric patients.

The enormous number of articles scattered across several databases can be somewhat overwhelming to practitioners. Therefore, this literature review was created to provide clinicians a summary of the available literature in hopes to aid in early identification of SNHL and/or ophthalmologic manifestations of various syndromes while also suggesting intervention strategies that may lead to better outcomes in child development and prevention of progressive loss of functioning.

The conditions involving both SNHL and ocular manifestations can be divided based upon involvement of pathology within the anterior segment, posterior segment, or both segments of the eye:

• Anterior Segment
• Posterior Segment
• Anterior and Posterior Segment

##  METHODS

Two authors (HAZ and DCP) conducted the literature search using the following databases: PubMed, Scopus, Elsevier, and Google Scholar was conducted to find relevant data published so far on ocular, auditory, and systemic manifestations of common pediatric syndromes. Search keywords included are *hearing loss, sensorineural, syndromes, ocular and auditory manifestations, Cogan Syndrome, Usher Syndrome, Hereditary Optic Nerve Neuropathy, Leber Hereditary Optic Neuropathy, Dominant Optic Atrophy, Wolfram Syndrome, Norrie Disease, Stickler Syndrome, Marfan Syndrome, Rubella, Alport Syndrome, Waardenburg Syndrome, Keratitis-ichthyosis-deafness syndrome, Heimler Syndrome, Cogan syndrome and vision, Usher syndrome and vision, Hereditary Optic Nerve Neuropathy and vision, Leber Hereditary Optic Neuropathy and vision, Dominant Optic Atrophy and vision, Wolfram Syndrome and vision, Norrie disease and vision, Stickler syndrome and vision, Marfan syndrome and vision, Rubella and vision, Alport* *syndrome and vision, Waardenburg syndrome and vision, Keratitis-ichthyosis-deafness syndrome and vision, Heimler syndrome and vision*. The retrieved articles were initially screened by title, and the articles with relevant titles were screened via abstract using predefined inclusion and exclusion criteria. Relevance of the articles were sometimes unclear from the abstract; thus, full articles were examined in these cases. Inclusion criteria included articles that were available in English and concerned sensorineural and/or visual manifestations for the disease entities studied in the article. Exclusion criteria included hearing loss that was not classified as sensorineural as well as the article not clearly discussing the aspects of the clinical syndrome (i.e., inheritance, pathophysiology, symptoms, management, or treatment). Because many of the aforementioned keywords yielded a significant number of articles, an informal literature search was conducted to identify articles that specifically met our inclusion and exclusion criterion – a total of 174 articles were ultimately included in this literature review. Searches were not restricted to ocular manifestation in patients with SNHL with a certain age range; however, a majority of the syndromes are diagnosed in childhood/adolescence, making it the primary age range studied by default. Relevant articles from each database were compiled with duplicates being removed. We categorized each article based upon the disease entity it described. The relevant literature in this review includes articles published from 1965–2021, with the majority of articles analyzed from 2010 to 2022.

##  DISCUSSION

### Anterior Segment Pathologies

#### Keratitis–ichthyosis–deafness (KID) syndrome

KID syndrome is a rare, congenital syndrome of the ectoderm that consists of the clinical trial of progressive vascularizing keratitis, SNHL, and skin manifestations.^[12]^ It has been reported to occur in both an autosomal dominant and recessive manner caused by a heterozygous mutation in the *GJB2* gene that encodes for connexin-26 on chromosome 13q12.^[13]^ KID syndrome was first proposed by Skinner et al^[14]^ and first described via familial occurrence by Grob et al^[15]^ in a father and daughter. Nazzaro et al^[16]^ also reports a case of a mother and daughter with KID syndrome, both of which presented with papillomatous hyperkeratosis and keratitis with corneal neovascularization. Corneal vascularization is present in up to 80% of cases. Additional corneal manifestations include corneal erosions and scarring, corneal leucomae, meibomitis, and severe dry eye. Messmer et al^[17]^ describes three patients in which visual acuity ranged from normal to severe visual loss and presented with ocular signs of eyebrow and lash loss, thickened and keratinized lids, trichiasis, recurrent corneal defects and vascularization, keratoconjunctivitis. Hearing loss in KID is typically a congenital form of SNHL but diagnosis is often delayed into infancy or early childhood which results in development delay, particularly in speech.

At birth, patients can present with generalized erythroderma as well as scaling and leathery skin. As children age, erythrodermic plaques can present on the body, especially in flexures of elbows and knees.^[13,18]^ The classic dermatologic findings include vascularizing keratitis and erythrokeratoderma; however, literature has shown the presence of a follicular occlusion triad with hidradenitis suppurativa, acne conglobate, and dissecting cellulitis of the scalp.^[19,20]^ Keratotic hyperplasic and inflammatory nodules can present on the body, which can later result in squamous cell carcinoma arising within these lesions.^[21,22]^

Management of KID involves a multidisciplinary approach targeting ophthalmologic, otolaryngologic, and dermatologic symptoms.

To prevent dry eye artificial and lubricating drops or anti-inflammatory agents can be very useful (Marshall). Treatment of corneal neovascularization typically involves topical steroids (i.e., predinosolone, dexamethasone) and anti-VEGF agents (i.e., bevacizumab) through antiangiogenic effects.^[23]^ Laser ablation and photodynamic therapy are more invasive procedures that can be considered for the management of corneal neovascularization in refractive cases.^[23,24]^

Cochlear implants in KID syndrome have been reported in multiple cases;^[25,26]^ however, it is important to consider that hearing assessment and aid fitting may be complicated by ichthyotic involvement of the ear canal. Barker and Briggs^[26]^ also discuss eczematous dermatitis and otitis media as an additional problem that may arise with cochlear implantation. Dermatological symptoms of hyperkeratosis can be managed with acitretin therapy while isotretinoin has variable efficacy for management of follicular occlusion.^[12]^ Systemic retinoids have been used with variable response and also pose potential ocular and skeletal toxicity.^[27]^ Although there is a good general prognosis, it is important to maintain life-long management and follow-up of patients due monitor malignancy and progression.

### Posterior Segment Pathologies

#### Usher Syndrome

Usher Syndrome (USH) is a rare, inherited disorder characterized by a constellation of neurological, auditory, and ophthalmic features. Worldwide the prevalence is noted to be between 4 and 17 in 100,000.^[28,29,30]^ The prevalence of USH in the United States is 4.4/100,000.^[31]^ It is inherited in an autosomal recessive fashion and is the most common cause for hereditary deafness and blindness in children, constituting 5% of congenital deafness and 18% of all retinitis pigmentosa patients.^[28,32]^ The major genes affected include *MYO7A* (USH1B), *USH1C*, *CDH23*, *PCDH15* (USH1F), *USH2A*, and *USH3A*. The Usher genes encode a variety of proteins that are expressed in the inner ear and retina, where they perform essential functions in sensory hair cell development and function as well as photoreceptor maintenance.^[6]^ Myosin VIIa and *USH2* genes are involved in visual pigment regeneration and maintaining photoreceptor health. Defects in these genes affect the transport of proteins, rhodopsin, and photoreceptors in retinal pigment epithelial cells.^[6,33]^ USH is considered to be a part of a group of disorders referred to as ciliopathies. Ciliopathies are characterized by abnormal formation or function of cilia, which are the integral structural components of almost all cells. Defective mutations can lead to abnormal proteins affecting the cilium–centrosome complex and, in turn, affecting the cellular signaling pathways. The major organs affected in ciliopathies include the retina, renal tissue, and cerebrum. Other manifestations include obesity, diabetes, skeletal dysplasias, and congenital fibrocystic liver diseases. Major ciliopathic syndromes include Alström syndrome, Bardet-Biedl syndrome, USH, Joubert syndrome, Leber congenital amaurosis, Nephronophthisis, Orofaciodigital syndrome 1, Polycystic kidney disease, and Meckel-Gruber syndrome.^[34,35,36]^

The characteristic clinical features of USH include RP, SNHL, and vestibular disturbances.^[37,38]^ Based on the onset and severity of hearing loss, vestibular disturbances, and RP, USH has been sub-grouped into three types. USH type 1 patients will have severe sensorineural deafness from birth, vestibular abnormalities and RP by the first decade. USH type 2 patients will have RP within the second decade of life, moderate to severe congenital hearing loss and no vestibular abnormalities. USH 3 patients will have progressive and variable hearing loss, vestibular abnormalities and RP.^[6][28,32]^ Despite this subtype classification, overlap of features and atypical presentations are also noted.

Children with USH typically present with bilateral SNHL and progressive retinal degeneration. The onset varies with each subtype. USH type1 children are born with profound hearing loss and early vision loss. They will also have severe balance abnormalities. Hence, USH type 1 children need early cochlear implantation to restore the hearing and early visual rehabilitation for RP. USH 1 is the most severe subtype among all.^[28,32][39][40]^ USH 2 subtype is the most common form and presents with moderate hearing loss and RP without balance abnormalities. USH type 3 has variable hearing, visual and balance abnormalities.^[38,41]^ Children with USH type 3 usually are not congenitally deaf. Most of the clinical features of USH type 3 will occur in the first or second decade and progress variably. Children with USH will have typical RP features including the onset of night blindness and peripheral visual field deterioration. Typical fundus signs include optic disc pallor, retinal arteriolar attenuation, and degenerative changes of retinal pigment epithelium.^[42,43,44,45]^

Management of USH includes early screening to detect the hearing and visual loss, thereby early initiation of therapy. Many children with USH may learn sign language due to hearing loss and later lose the same art due to progressive visual loss; hence, audiological rehabilitation is crucial for all subtypes of USH with cochlear implants or hearing aids. Visual rehabilitation and low vision aids services have to be provided for progressive visual loss due to RP.^[46,47]^ Treatment directed toward the definitive gene therapy is ongoing. Thus far, genetic counselling, risk assessment, and supportive therapies are the mainstay of USH management.

### Hereditary optic nerve neuropathies

#### Autosomal Dominant Optic Atrophy

Autosomal Dominant Optic Atrophy (ADOA) is the most common hereditary optic neuropathy. It is caused by a mutation in the *OPA1* gene, which is the most common gene involved in the development of ADOA. OPA1 encodes for a mitochondrial dynamin-related GTPase responsible for maintaining the mitochondrial membrane.^[48]^ Loss of function of this GTPase can lead to mitochondrial function due to loss of mitochondrial fusion and oxidative phosphorylation with subsequent build-up of reactive oxygen species and altered calcium homeostasis, all of which lead to retinal ganglion cell apoptosis.^[48,49]^

ADOA is found in males and females equally and commonly presents in school-aged children due to complaints of decreased visual acuity in both eyes. There is a gradual progression of bilateral vision loss with central or cecocentral scotoma field defect as well as loss of color vision.^[50]^ The majority of patients present with visual acuity < 20/200, with some patients having asymmetric visual function (i.e., varying visual acuity in the right and left eyes).^[50,51]^ The predominant finding on fundoscopy is optic atrophy. OCT imaging is significant for pallor limited to the temporal portion of the disc [Figure 2], indicative of papillomacular bundle nerve fiber loss. ADOA can be confused with glaucoma when analyzing imaging, thus ADOA is commonly referred to as “pseudo-glaucoma”.^[49,51,52]^ Imaging consistent with glaucoma shows reduced cupping with a cup-to-disc ratio > 0.5.^[52,53]^ Additionally, peripapillary atrophy is seen similar to that in glaucoma. However, through analysis of other clinical findings and pertinent imaging findings, ADOA can be distinguished from glaucoma. These findings include presentation in childhood, normal intraocular pressure (IOP), central/cecocentral scotoma, temporal rim pallor, and saucerization of the optic disc.^[48,53]^

The findings of dominant optic atrophy have also been reported with other systemic manifestations, termed Autosomal Dominant Optic Atrophy plus syndrome (“ADOA plus”). Additional features of this syndrome are significant for SNHL, vestibular dysfunction, ataxia, external ophthalmoplegia, and muscle myopathy.^[54,55,57]^ Yu-Wai-Man et al^[54]^ found that SNHL was the most common manifestation in patients with ADOA plus syndrome alongside optic atrophy. These systemic features may present after the ophthalmic manifestations; thus, it is important to follow-up with these patients as they age.^[54]^ There is no current treatment for ADOA, but nutritional vitamin supplements such as vitamin B12, C, and folate are commonly used to reduce stress on the optic nerve due to reactive oxygen species.^[58]^ Other forms of management include Coenzyme Q, which is also used for its antioxidant properties.

Idebenone has been found to stabilize/aid in recovery of patients' visual acuity; however, there are still concerns regarding its mechanistic effects.^[59,60]^ Santarelli et al^[61]^ demonstrate with increasing age, hearing threshold deteriorates and speech perception difficulty increases, making it imperative for patients to be monitored through audiometry tests and auditory brain response tests. Patients should be regularly followed by their ophthalmologist and a retina specialist. Other members of the family should also be prompted to get screened due to the hereditary nature of this disease.

#### Wolfram Syndrome

Wolfram Syndrome (WFS) is a rare, neurodegenerative syndrome caused by a mutation in the *WFS1* or *WFS2* gene. WFS has been termed by the acronym “DIDMOAD” to signify the manifestations of diabetes insipidus, diabetes mellites, optic atrophy, and deafness.^[62,63,64]^ It has an estimated prevalence of 1 in 100,000 in the United States and 1 in 150 cases of juvenile-onset insulin-dependent diabetes.^[62,65]^ WFS is inherited in an autosomal recessive pattern; however, patients with the *WFS1* gene mutation inherit several pathologies in an autosomal dominant manner. These include a low-frequency SNHL, optic atrophy, type 2 diabetes, and psychiatric problems (i.e., depression and anxiety).^[64,66,67,68]^ WFS can also present with several neurological impairments, such as peripheral neuropathy, cerebellar ataxia, and myoclonus.^[69]^ Initial presentation of WFS usually begins with the onset of diabetes in the first decade of life and optic atrophy, diabetes insipidus, and deafness during the second decade.^[64,70]^ Patients who experience neurological impairments and genitourinary pathology, such as urinary incontinence, have onset of these symptoms during their third decade of life.^[64]^

Requirements for the diagnosis of WFS include juvenile diabetes mellitus and optic atrophy. The most common ocular manifestation is a progressive optic atrophy, with a median age of 11 years.^[71]^ The symptoms of optic atrophy typically present as decreased visual acuity, central scotoma, and loss of color vision in the blue–yellow spectrum.^[71,72]^ Other ocular findings may include cataracts, pigmentary retinopathy, diabetic retinopathy, and nystagmus.^[73]^ With progression of visual loss, loss of pupillary response to light is also seen.^[69,71]^ Like LHON and DOA, WFS may be associated with retinal ganglion abnormalities; studies investigating electrophysiology, cranial MRI imaging, and postmortem examination of patients with WFS have found that the optic nerve is the main site of neurodegeneration.^[71,72]^ Optical Coherence Tomography-Angiography (OCT-A) of patients with WFS has shown similar findings to that seen in LHON, such as radial peripapillary capillary and retinal nerve fiber layer thinning as well as involvement of the microvasculature of the papillomacular bundle.^[74]^ Approximately 60% of patients with WFS also develop SNHL by the second decade of life.^[75]^ With increasing age, hearing loss becomes more pronounced with progressive neurodegeneration.

The prognosis is poor with death commonly occurring near the end of the third decade of life. The main cause of death usually results from progressive neurodegeneration and brainstem atrophy, resulting in respiratory failure.^[76]^ Due to the many organ systems involved in this disease it is imperative to approach consistently monitor each pathology. Endocrinologists, audiologists, and ophthalmologists should all be involved in the care of the patient with the aim to slow the progression of the disease through constant monitoring and therapeutic strategies. Hearing loss can be monitored through audiometry tests and auditory brain response test to monitor the course of disease. Ocular manifestations can be monitored by routine fundoscopy, OCT imaging, and MRI. No cure is currently available for WFS patients but therapy with medications to treat each individual pathology (i.e., glucose control and insulin therapy to treat diabetes mellitus). Treatment with hearing aids or cochlear implants is an option for patients with SNHL.^[76,77]^ Clinical trials examining the efficacy of idebenone and docasahexenoic acid have been shown to slow the progression of optic atrophy.^[78]^

#### Leber Hereditary Optic Neuropathy

Leber Hereditary Optic Neuropathy (LHON) is an inherited mitochondrial disorder characterized by degeneration of the optic nerve. LHON is caused by mutations in the *MT-ND1*, *MT-ND4*, *MT-ND4L*, and *MT-ND6* genes.^[79,80,81,82]^ NADH dehydrogenase is part complex I in the electron transport chain, important for producing ATP. These mutations result in oxidative stress and damage to the optic nerve. Mutations in the glutamate transport system have also shown to contribute to oxidative stress and dysfunction of the ganglion cells of the retina.^[81,83]^ Men aged 20–30 are most affected, commonly presenting with bilateral, painless and progressive vision loss.^[84]^ It is thought that environmental factors, such as smoking and alcohol intake, may play a role in the expression of disease.^[85]^ Patients initially present with visual loss in one eye that later progresses to bilateral visual loss. The involvement of both eyes can be simultaneous or sequential, occurring months to years following the initial unilateral visual loss.^[86,87]^ Fundoscopy of LHON may be normal or show hyperemic optic nerves with peripapillary telangiectasias [Figure 3]. Similarly, OCT-A is significant for involvement of microvasculature of the small axons of the papillomacular bundle, radial peripallary capillary defects, and a decrease in the retinal nerve fiber layer with progress of the optic atrophy.^[88]^. Patients may experience loss of color vision.^[84,89]^

LHON can also manifest systemic symptoms, classified as “Leber's plus”, with decreased ability to control muscle movements, tremors, and cardiac arrhythmia; this has been compared to multiple sclerosis (MS) due to its mitochondrial inheritance as well as similar white matter brain lesions seen on MRI.^[90,91]^ Auditory dysfunction has been noted in case studies that found patients, particularly those containing the *mt11778* mutation, showing impaired detection of auditory cues and abnormal speech understanding.^[82]^. However, this form of auditory loss differs from other forms of SNHL due to normal cochlear outer hair cells.^[84,86]^

Treatment with antioxidants has been used to decrease reactive oxidation. Specifically, idebenone has been studied.^[58,92,93]^ Additionally, gene therapy has shown promising progress toward using adeno-associated viral vectors for monogenic blinding diseases, like LHON. A study conducted by Sahel et al^[94,95]^ found that transfer bilateral improvement in visual function with the use of a viral vector DNA injected into subjects with LHON. Patients should be advised to refrain from smoking and to limit their alcohol intake due to evidence of increased visual impairment.^[85]^ DNA testing can confirm mitochondrial gene mutations.

### Marfan Syndrome

Marfan Syndrome (MFS) is a multisystem autosomal dominant connective tissue disorder caused by a mutation in fibrillin-1 (*FBN1*) gene on chromosome 15. *FBN1* gene encodes fibrillin 1, a structural component of the extracellular matrix (ECM) and also involved in regulation of transforming growth factor β (TGF-β).^[96,97,98]^ Incidence of MFS varies between 1/5,000 and 1/20,000.^[99]^ Major organ systems involved are cardiovascular, pulmonary, ocular, and skeletal.^[100]^ There are various clinical criteria for diagnosis of MFS like clinical criteria by Beighton, Ghent-1, and Ghent-2.^[101,102,103]^ The revised Ghent-2 criteria is more simplified for diagnosis and is characterized by three clinical criteria (i.e., thoracic aortic aneurysm and/or dissection, ectopia lentis, and systemic features) and two genetic criteria (i.e., the presence of a first-grade relative with MFS and presence of a pathogenic mutation in FBN).

Systemic features of MFS include cardiovascular anomalies (e.g., mitral valve prolapse, aortic aneurysm, aortic dissection), pulmonary involvement (e.g., pneumothorax and apical lung blebs), and musculoskeletal features (e.g., tall and thin built, scoliosis,

pectus excavatum, high-arched palate, facial abnormalities and flexible extremities with arachnodactyly). Often severe cardiovascular complications lead to early mortality in MFS patients.^[96,97,104]^

Hearing loss is often seen in children and young adults with MFS. Skeletal derangement which leads to hearing loss includes long narrow face and skull, large low-set and posteriorly rotated ears, narrow and angulated ear canals, and ossicular malformation. All these structural anomalies will lead to congenital hearing loss, chronic otitis media, eustachian tube dysfunction and lead to SNHL.^[105,106]^

Ophthalmologic features of MFS include bilateral ectopia lentis, myopia, amblyopia, strabismus, keratoconus, hypoplastic iris with miosis and retinal detachment. Ectopia lentis, myopia, and retinal detachment being the most common and major ocular features of concern.^[107]^ Lens subluxation is often bilateral (50–80%); the most common location is superotemporal subluxation. It can present in early life in the first or second decade of life. The lens can also get dislocated into the anterior or posterior chamber, causing secondary uveitis. Occurrence of myopia is common with increased axial length of the eyeball. Due to ectopia lentis and myopia, patients will have blurred vision, monocular diplopia, astigmatism and amblyopia. High myopia with an increased length of the globe predisposes to vitreous liquefaction, lattice degeneration, and retinal tears eventually increasing the risk of retinal detachment. About 5–26.5% of MFS patients develop retinal detachment often bilaterally in 30–40% of cases.^[99,108]^

The management of MFS includes regular screening of MFS patients for systemic, ocular features, and addressing the complications associated with them. Prophylactic β-blockers and angiotensin II-receptor blockers can slow down the dilation of the ascending aorta, and prophylactic aortic surgery when needed. β-blocker therapy may reduce TGF-β activation, which has been recognized as a contributory factor in MFS. Early audiologic screening is mandatory to identify the cause of hearing loss and address it.^[96][105,106]^

Early correction of refractive error helps in preventing anisometropia and amblyopia. Mild ectopia lentis can be addressed with glasses, whereas severe dislocation with complications will require surgical removal of the lens and placement of intraocular lens.^[109]^

Prophylactic barrage laser for retinal lattice degeneration and retinal tears can prevent occurrence of retinal detachment. Once retinal detachment develops, early vitreoretinal surgery is indicated for preserving the vision.^[110,111]^

A close follow-up of all MFS patients with a multidisciplinary approach involving cardiologist, orthopedician, otolaryngologist, and geneticist is mandatory for better morbidity and to prevent mortality.

### Norrie Disease

Norrie disease (ND) is a rare genetic disorder which is X-linked recessive. *Norrie disease protein* (*NDP*) gene mutation located on the short arm of the X chromosome (Xp11.3) leads to defective norrin.^[112,113]^ More than 100 pathogenic variations of *NDP* gene have been reported with most common being contiguous deletions. Complete penetrance is seen in affected males whereas females remain unaffected carriers.^[114,115]^ The function of norrin is normal angiogenesis in particular retinal vasculogenesis and development of inner ear cells. The defective norrin protein leads to retinal dysgenesis, disorganized tissues and fibrovascular proliferations.^[116,117]^

ND patients will have classic triad of blindness from birth or as neonates, progressive hearing loss in adolescence, and cognitive or behavioral problems.^[112]^ Ophthalmologic manifestations are the first and earliest sign of ND in any male child and will be present in almost all children. The cause of blindness at birth is due to immature and dysgenic retinal cell masses referred to as pseudogliomas and retinal detachments, which can occur in utero. ND is one of the important differentials for leukocoria at birth or in neonates. Other ophthalmic features are microphthalmia, atrophic iris, synechiae, angle abnormalities, increase in intraocular pressure, glaucoma, and cataract. At the end stage of the disease corneal opacification, calcific band keratopathy, and phthisis bulbi are noted.^[112,113][118]^

SNHL is a common finding due to loss of vessels in the stria vascularis of the cochlea. It has also been reported that approximately all patients with ND will have some degree of hearing loss in their lifetime. Early hearing symptoms are tinnitus or stuffiness. By adulthood, hearing loss progresses with patients having bilateral deafness in severe form.^[119,120]^

Developmental delay, intellectual disability, autism, depression, and psychotic features are the major cognitive abnormalities seen in about 30–50% of males with ND. Rarely, severe infantile spasms and chronic seizures have been reported.^[121]^ Other clinical features of ND include peripheral vascular diseases like venous stasis ulcers, varicose veins, and erectile dysfunction. These features mostly occur in older ages beyond 30–50 years.^[122]^

ND is mostly a clinical diagnosis by ophthalmic, hearing, and neurodevelopmental assessment. However, the definitive diagnosis is by genetic analysis for the presence of a pathogenic variant in the *NDP* gene. The management is a multidisciplinary approach involving ophthalmologists, otolaryngologists, and neurologists. Unfortunately, patients presenting with complete retinal detachments at birth do not benefit with any surgical intervention. For patients with partial retinal detachments and abnormal vascularization, vitreoretinal surgery and laser photocoagulation therapy will help in preventing blindness.^[123,124]^ Visual rehabilitation and low vision aids are mainstay for patients of ND with poor vision. For hearing abnormalities, assistive devices and cochlear implants for severe cases are helpful.^[120]^ Supportive therapy is recommended for behavioral and cognitive abnormalities. Genetic counselling of parents with genetic risk assessment for future generations is crucial to prevent the occurrence of ND in other family members.

### Stickler Syndrome

Stickler syndrome is a hereditary connective tissue disorder due to defects in collagen production. Major collagen types affected are type II, IX and XI.^[125,126,127]^ It is inherited in an autosomal dominant pattern with the most common defect in the gene *COL2A1*. The subtypes are based on ocular and systemic features with a specific genetic defect. Type 4 has only ocular features.^[127,128]^

Almost all patients with ocular involvement will present with vitreous syneresis, beaded vitreous, and radial perivascular retinal lattice degeneration.^[127,129]^ Other ocular features are myopia, lamellar cataract, open-angle glaucoma, megalophthalmos, and anterior segment dysgenesis. It is the most common cause of rhegmatogenous retinal detachment in childhood due to the formation of giant retinal tears. Lamellar cataract in most cases is peripheral and does not always effect the visual function. The ocular features start in early childhood within 4–6 years of age.^[129,130,131,132]^ Hearing abnormalities in stickler syndrome are either sensorineural or conductive. Collagen affection in the cochlea, middle ear, and tympanic membrane is causative for hearing loss. Regular audiometric check-up is recommended for early detection of hearing loss in stickler syndrome.^[133,134]^

Patients with stickler syndrome typically present with the Pierre-Robin sequence which includes small mandible, retraction of the tongue, upper airway obstruction, and cleft palate. Other systemic features include hypermobility and osteoarthritis of the knee, hip and spine.^[126,128,130]^

Diagnosis of the syndrome is mainly clinical, depending on the spectrum of clinical manifestation. Molecular genetic testing can be performed for confirmation of diagnosis.^[126,128]^

Management of stickle syndrome includes regular screening for the systemic as well as ocular and auditory features. Early detection with fundus examination, audiometry, and radiographic investigation will help in early definitive and supportive therapy. Ophthalmic features of significant cataract and retinal detachment are treated surgically. Prophylactic laser barrage for the lattice degeneration and retinal tears helps in prevention of retinal detachment. Glaucoma is managed medically initially. Uncontrolled glaucoma with topical medications is managed surgically with trabeculectomy, goniotomy, or filtering procedures.^[132,135]^ Hearing loss is managed by digital hearing aids or cochlear implantation. Surgical intervention involving the middle ear has been reported in few studies.^[126,133]^ Musculoskeletal abnormalities are managed conservatively with pain management in acute conditions and supportive therapy with exercise to maintain stability in chronic conditions. Surgical intervention is recommended for disability to replace or re-align joints.^[126,127]^ Overall, early screening, prompt referral to specialist physician, genetic counselling to reduce the risk occurrence in future family members and supportive therapy are crucial in the management of stickler syndrome.

### Heimler Syndrome

Heimler Syndrome (HS) is a rare, autosomal recessive disorder characterized by SNHL, amelogenesis imperfecta, nail abnormalities, and retinal dystrophy (RD). HS is a form of peroxisomal biogenesis disorders and has been found to involve biallelic variations in peroxismal biogenesis factors 1 (*PEX1*) and 6 (*PEX6*).^[136]^ HS was first described by Heimler et al in a brother and sister who presented with SNHL, enamel hypoplasia, and nail abnormalities.^[137]^ Few cases have been reported to date; however, Mechausseir et al propose that HS is most likely underdiagnosed due to misdiagnosis of enamel defects.^[136]^ Ophthalmologic manifestations of HS include RD, which can present retinitis pigmentosa and macular degeneration. RD was not reported in the original description of this disease by Heimler et al, however, in 2011 Lima et al reported a 29-year-old woman with HS who developed bilateral vision loss.^[137,138]^ It was hypothesized that HS is possibly an expression of a ciliopathy that can result in RD. The presence of RD can depend on the age of the patient, with earlier onset of diagnosis more commonly found. All patients with HS described in the literature have presented with congenital SNHL. This is typically found at birth bilaterally but can rarely have a unilateral presentation. This is initially found when newborns fail their hearing screen and is typically the first diagnostic clue. Due to the presence of both SNHL and retinal pigmentation, HS is important to consider within the differential diagnosis of ciliopathies, like US described previously. Other presentations of HS include nail abnormalities, which involve nail ridging (i.e., Beau's lines) and punctate leukonychia, which can present on both finger and toenails.^[137,139]^ Enamel hypoplasia presents as yellow–brown discoloration of teeth with a granular appearance and affects only the permanent secondary dentition. Premolar and molar teeth are commonly affected in a more severe fashion compared to anterior teeth.^[137,139][140][141]^

A thorough systemic evaluation is necessary for patients with suspected HS. The initial presentation is most commonly hearing loss found on newborn exam, it is important to follow-up with audiologic and otolaryngologic management to ensure proper development of speech and evaluation for cochlear implant surgery. For patient with RD, management depends on the underlying disease process. There is no specific treatment of retinitis pigmentosa; however, vitamin A supplementation has been shown to slow the progression of vision loss.^[142]^ There is also no known cure for macular dystrophy; however, new research is being developed looking into gene and stem cell therapy.^[143,144]^ There is no standard of care for amelogenesis imperfecta; however, treatment typically involves good dental hygiene and can also involve bonding. Conservative or prosthetic and orthodontic treatment via the use of crowns should be strongly necessary for oral rehabilitation.^[145]^

### Anterior and Posterior Segment Pathologies

#### Cogan Syndrome

**Figure 1:**
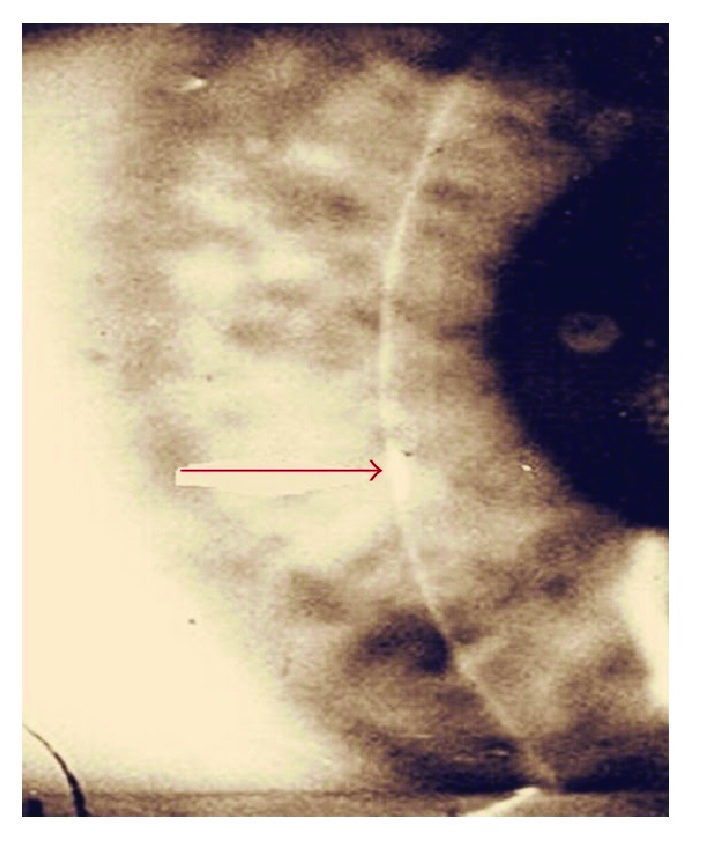
Slit-lamp examination of Cogan Syndrome**. **Interstitial Keratitis on slit lamp exam in a patient with Cogan Syndrome.^[204]^

Cogan Syndrome (CS) is a rare autoimmune vasculitis that classically affects young adults/adolescents but has no racial or sex predilection. CS was previously categorized based upon “typical” and “atypical” forms, defined by the presentation of ocular symptoms; however, this classification has been largely abandoned due to its limited prognostic significance.^[146]^ It is characterized by non-syphilitic interstitial keratitis (IK), intraocular inflammation, and vestibuloauditory dysfunction.^[147]^ Patients may initially present for complaints of eye redness, pain, visual loss, or photophobia; however, the predominant ocular finding of CS is IK. IK can be visualized on slit-lamp examination as an irregular, granular infiltration on the posterior aspect of the cornea^[148]^ [Figure 1]. Slit-lamp examination is notable for inflammation and crystalline deposits on the corneal stroma.^[149]^ Despite being known for the presentation of non-syphilitic IK, CS may present with a wide array of other inflammatory ocular manifestations such as episcleritis or scleritis, retinitis, conjunctivitis, and iridocyclitis.^[5,150]^ Due to the autoimmune nature of this disease, almost all patients will have an elevated WBC count and erythrocyte sedimentation rate (ESR).^[146,149]^

The vestibuloauditory symptoms of CS can present similarly to Meniere's disease with attack symptoms of tinnitus, nausea, and vomiting while patients may also present with bilateral, progressive hearing loss over months to years.^[146,151]^ Some patients may experience progressive bilateral hearing loss before ocular pathology presents, making it difficult to distinguish whether the

symptoms are stemming from a syndrome or a solitary inner ear disease. Systemic features of CS can involve other rheumatologic manifestations, such as vasculitis and aortitis.^[146,152]^ Cases have been reported of patients with CS having aortitis, aortic valve regurgitation, and right coronary artery stenosis.^[5,153]^

Diagnosis of CS is based upon clinical presentation of ocular symptoms and vestibuloauditory dysfunction. Patients may initially present with either ocular or auditory pathology, making it important for clinicians to investigate both systems to effectively diagnose and manage this progressive disease. The first-line treatment of CS includes systemic corticosteroids. Topical steroids are also used for treatment of IK. Other immunosuppressive agents have emerged including the use of tumor necrosis alpha inhibitors. Cochlear implantation is offered to patients who have irreversible SNHL. In patients with aortitis, aortic valve replacement may be necessary.

### Congenital Rubella

Congenital Rubella Syndrome (CRS), also commonly known as German Measles, is caused by a viral rubella infection of the fetus in utero. It is rare in developed countries with only a few cases per year due to immunization.^[154]^ Maternal infection during the first and third trimester is most harmful to the fetus—if the fetus survives infection in utero, it is likely they will be delivered preterm and/or have congenital defects.^[155]^ These congenital defects are widely known by the triad: cataracts or glaucoma, SNHL, and congenital heart disease.^[156]^ Another common manifestation includes CNS involvement, which may present with physical abnormalities of the skull (e.g., microcephaly, large anterior fontanelle) as well as motor, behavioral, or developmental delay. Other common manifestations include neurologic, hematologic, and endocrine abnormalities. These may include but are not exclusive to petechiae, hepatosplenomegaly, hemolytic anemia, jaundice, diabetes, among many others.

The most common ocular manifestations include cataracts, microphthalmia/microcornea, pigmentary retinopathy (i.e., “salt and pepper”), scleral jaundice, and glaucoma.^[155,157,158]^ Cataracts are the most predominant ocular manifestation, which is thought to occur due to viral entry into the lens in utero prior to a barrier formed by the lens capsule development. Bilateral cataracts are also commonly seen in comparison to a unilateral presentation. Delayed ocular presentations may occur, specifically with pigmentary retinopathy. In 40–60% of cases, “salt and pepper” retinopathy is visualized on fundoscopy as a speculated retina with hyper- and hypo-pigmented spots^[155]^ [Figure 4]. It is not commonly associated with visual loss; however, in some cases progression of pathology may occur leading to neovascularization, contributing to visual loss. Patients with “salt and pepper” retinopathy may commonly have sustained VA until later adulthood where they experience rapid visual loss due to neovascularization of the retina.

Bilateral SNHL is present in two-thirds of infants infected with rubella. Complete loss of hearing is common with progression of disease. The extent of hearing loss is not fixed, as CRS has been found to affect all frequencies uniformly.^[159]^

Diagnosis, per CDC guidelines, requires a rubella immunoglobulin M (IgM) antibody to be detected in serum in an infant < 6 months old, a sustained rubella immunoglobulin G (IgG) antibody level in serum in an infant between 6 and 12 months of age on ≤ 2 occasions with the absence of rubella vaccine or exposure to rubella, or a nucleic acid amplification test (NAAT) from a clinical sample (e.g., throat swab, nasal swab, blood). Despite this, it is important to consider that IgM levels may not be present at birth; thus, an infant who may be suspected to have CRS should be retested at one month of age or perform a NAAT if < 6 months. Pregnant females may also undergo rubella IgM testing if infection is suspected.^[156,160]^

Prenatal management is vital to reduce risk of infection to the fetus in utero. Mothers should be advised to receive the rubella vaccination prior to becoming pregnant. Similarly, all infants should be vaccinated after six months of age to prevent risk of infection later in life. Due to the systemic nature of CRS, multidisciplinary management is necessary from respective specialists.

Bilateral cataracts are a direct indication for surgical removal in infancy, which should be performed within the two to three months of life. Patients who present with unilateral cataracts may not meet requirements for surgery. Requirements VA 20/50 or worse, reduced visual response, opacity > 3 mm, and presentation of strabismus or nystagmus. Unfortunately, the surgical outcomes of infants with cataracts are poor because of severe inflammation.^[155]^ Patients with infantile glaucoma require surgical intervention. Surgical procedures are first line for infantile glaucoma and include goniotomy and trabeculotomy; second-line management includes draining devices.^[161]^ Other forms of management include medical therapy with carbonic anhydrase inhibitors, beta blockers, prostaglandin, alpha 2 agonists, or sympathomimetics. These medical options, however, are not preferred. Geniotomy and trabeculotomy have been shown to have the highest success rate at decreasing intraocular pressure.^[161,162]^ Infants with suspected or confirmed CRS should follow-up with a pediatric ophthalmologist to optimize visual care.

All infants, particularly those with suspected rubella infection in utero, must receive auditory evoked response testing after birth.^[159]^ This test is sensitive and specific, making it an important tool to diagnose SNHL and guide further management. Scheduled hearing assessments should occur during the neonatal period if patients are symptomatic. Management of hearing loss includes the use of hearing aids, bone conduction hearing devices, or cochlear implants. With greater severity of hearing loss, particularly in cases of bilateral SNHL, patients may require hearing cochlear implants.

**Table 1:** Summary of syndromes that include both ocular and sensorineural hearing loss manifestations.


**Syndrome**	**Age group affected**	**Sexpredilection**	**Prominent systemic manifestations**	**Ocular manifestations**	**Auditory** **manifestations**
**Alport Syndrome**	Early Childhood, Adolescence	Male	· Nephropathy^[169]^ · Hematuria^[10,165,170,175]^	· Anterior lenticonus^[7,171,172,173,190]^ · Posterior lenticonus^[168,171,190]^ · Cataracts^[168,171]^ · Macular flecked retinopathy^[171,174,190]^ · Nystagmus^[190]^ · Corneal erosion^[168,171]^ · Microcornea^[168,171]^ · Spontaneous lens rupture^[168,171]^ · Spherophakia^[168,171]^ · Posterior polymorphous corneal dystrophy^[171]^	· SNHL^[7,169]^ · Hearing loss may parallel severity of renal involvement^[190]^ · Cochlear lesions^[167]^
**Keratitis-Ichthyosis-Deafness Syndrome **	Infancy, Neonatal	No preference	· Erythroderma^[17]^ · Scaling and leathery skin^[17]^ · Eryhtrodermic plaques on flexure surfaces of body^[13,18]^ · Follicular occlusion triad^[19,20]^: Hidradenitis suppurativa Acne conglobate Dissecting cellulitis of the scalp · Keratotic hyperplastic and inflammatory nodules^[21,22]^ · Squamous cell carcinoma^[21,22]^	· Corneal vascularization 12, 16, 17, 23 · Corneal erosions and scarring 12 · Corneal leucomae^[23,191]^ · Meibomitis^[12,191]^ · Dry eye^[12,191]^ · Eyebrow and lash loss^[17]^ · Thickened and keratinized lids^[17]^ · Trichiasis^[17]^ · Keratoconjunctivitis^[17,191]^	· SNHL17, 22, 27, 191
**Waardenburg Syndrome**	Infancy, Neonatal	No preference	· Pigmentation anomalies^[181,184,185]^ · Poliosis^[184]^ · Broad nasal root^[187,188]^ · Synophrys^[185]^	· Iris Heterochromia^[181,185,186,188]^ · Ptosis^[184,185]^ · Strabismus^[185]^ · Choroidal Hypopigmentation^[185,186,188]^ · Dystopia canthorum^[181,188]^ · Hypertelorism, Broad nasal root^[181,184]^	· Predominantly bilateral SNHL^[192]^
**Norrie Disease**	Infancy, Neonatal	Male	· Cognitive impairment^[112]^ · Psychomotor retardation^[121]^ · Autism Spectrum Disorder · Seizure Disorder^[121]^ · Hypertelorism^[193]^ · Narrow nasal bridge^[193]^	· Congenital blindness^[112,194]^ · Retinal dysgenesis^[116,117]^ · Retinal detachment^[123]^ · Pseudoglioma^[112,195]^ · Cataracts^[74,194]^ · Corneal opacities^[112,113][118]^ · Bulbar atrophies^[112,113][118]^ · Microphthalmia^[196]^ · Iris hypoplasia^[74]^ · Phthisis bulbi^[118]^	· SNHL^[192]^ · Vascular abnormalities of the cochlea^[116,117][122]^
**Heimler Syndrome**	Infancy, Neonatal	No preference	· Nail abnormalities^[136][137,139][140][141][197][198]^(i.e., nail ridging, punctate leukonychia) · Amelogenesis Imperfecta/Enamel hypoplasia^[136][137,139][140][141][197]^	· Retinal dystrophy (i.e., retinitis pigmentosa, macular dystrophy)^[136,138,139,197,198]^	· Unilateral or bilateral SNHL^[136][137,139][140][141]^
**Cogan Syndrome**	Young Adulthood ( ∼ 25 yr)	No preference	· Vasculitis^[146,152]^ · Aortitis^[5][146,152][153]^ · Aortic Valve Regurgitation^[5,153]^ · Coronary Artery Stenosis^[5,153]^	· Interstitial keratitis^[147]^ · Iridocyclitis^[5,150]^ · Scleritis^[5,150]^ · Episcleritis^[5,150]^ · Conjunctivitis^[5,150]^ · Retinal artery occlusion^[199]^ · Choroiditis^[199]^ · Retinal hemorrhages^[199]^ · Papilledema^[199]^ · Exophthalmos^[199]^	· SNHL^[5,6,11]^
**Usher Syndrome**	Infancy, Neonatal	No preference	· Ciliopathies^[34,35,36]^	**USH1** · Onset of Retinitis Pigmentosa (RP) in 1st decade of life^[6][28,32][41]^ **USH2 ** · Onset of RP within 2nd decade of life^[6][28,32][41]^ **USH3** · Onset of RP is progressive, sporadic, and variable^[6][28,32][41]^	**USH1** · Congenital severe-to-profound deafness^[6][28,32][41]^ · Vestibular areflexia^[6][28,32][38]^ **USH2** · Congenital moderate-to-severe hearing loss^[6][28,32][38]^ **USH3** · Progressive and variable vestibular hearing loss^[28,32][38]^
**Stickler Syndrome**	Young Adulthood	No preference	· Pierre-Robinson Sequence: small mandible, retraction of the tongue, upper airway obstruction, and cleft palate^[11][126,128][133]^ · Hypermobility^[126,128,130]^ Early onset osteoarthritis^[126,128,130]^	· High myopia^[128]^ · Vitreous abnormalities^[127,129]^ · Retinal detachment^[11][126][127,129][200]^ · Glaucoma^[132,135]^ · Cataracts^[128,132]^	· SNHL^[120,121]^
**Autosomal Dominant Optic Atrophy**	Adolescence, Young Adulthood	No preference	· Movement Disorders^[53][54,55,57]^ · Peripheral neuropathy^[201]^ · Myopathy^[201]^	· Optic Atrophy^[8,9,49,51,56]^ · Progression of bilateral vision loss with central or cecocentral scotoma^[50]^ · Loss of color vision (blue-yellow spectrum)^[50]^	· SNHL^[8,9,49,51,56]^
**Wolfram Syndrome**	Adolescence, Young Adulthood	No preference	· Diabetes Inspidus^[64,70]^ · Diabetes Mellitus^[64,70]^ · Peripheral Neuropathy^[69]^ · Cerebellar Ataxia^[69]^ · Myoclonus^[69]^ · Urinary Incontinence^[64]^	· Optic Atrophy^[69]^ · Progressive bilateral vision loss^[69,71]^ · Loss of color vision (blue-yellow spectrum)^[71,72]^ · Cataracts^[73]^ · Pigmentary retinopathy^[73]^ · Nystagmus^[73]^	· SNHL^[51,53,54,55]^
**Leber Hereditary Optic Atrophy**	Adulthood	Males	· Decreased muscle control^[90,91]^ · Tremors^[90,91]^ · Cardiac arrhythmia^[90,91]^	· Optic Atrophy^[88]^ · Progressive bilateral vision loss^[86]^ · Loss of color vision (red-green spectrum)^[84,89]^	· SNHL^[82,71,73]^
**Marfan Syndrome**	Infancy, Neonatal	No preference	· Aortic root dilation^[96,97,104]^ · Mitral valve prolapse^[96,97,104]^ · Aortic aneurysm^[96,97,99,100,202]^ · Aortic Dissection^[96,97,99,100,202]^ · Pneumothorax^[96,97,99,202]^ · Tall, thin habitus^[83,98]^ · Scoliosis^[83,98]^ · Pectus excavatum^[83,85]^ · High-arched palate^[83,98]^ · Facial abnormalities^[105,106]^ · Flexible Extremities^[98]^ · Arachnodactyly^[83,85]^	· Amblyopia^[108,203]^ · Keratoconus^[108]^ · Coloboma^[107]^ · Myopia^[107]^ · Lens dislocation^[107]^ · Retinal detachment^[107,203]^ · Strabismus^[203]^	· SNHL with high rates of congenital hearing loss^[92,93]^ · Chronic otitis media^[105]^ · Eustachian tube dysfunction^[105]^
	
	
(Azami A, Maleki N, Kalantar Hormozi M, Tavosi Z. Interstitial keratitis, vertigo, and vasculitis: Typical Cogan's Syndrome. Case Rep Med 2014;2014:830831)					

**Figure 2:**
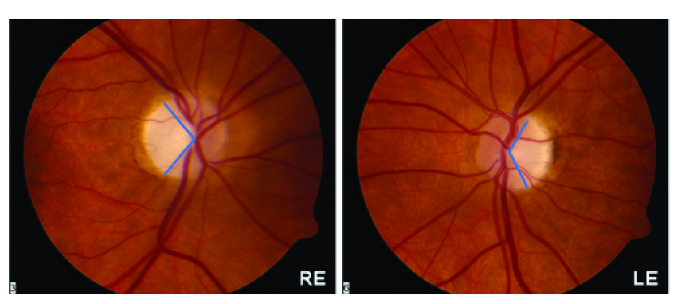
Temporal pallor seen in dominant optic atrophy. Both eyes optic disc photograph (RE – right eye, LE – left eye) showing temporal pallor with loss of fine capillary network in a case of Dominant Optic Atrophy with *OPA1* mutations.^[54]^

**Figure 3:**
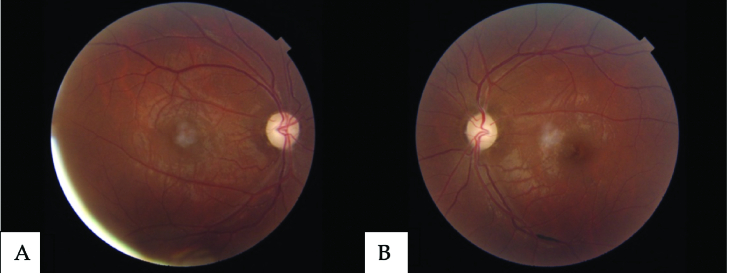
Fundus images of Leber hereditary optic neuropathy. Fundus photographs of a patient with Leber hereditary optic neuropathy revealing optic nerve pallor on both eyes ([A] right eye and [B] left eye).

**Figure 4:**
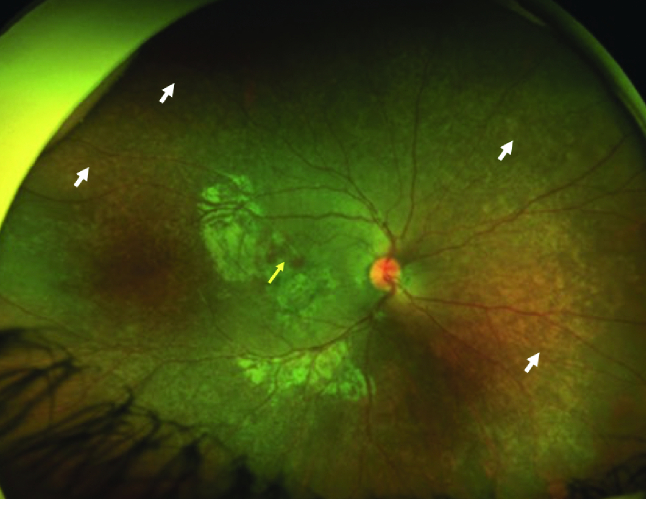
Fundus image of congenital rubella syndrome.Wide field color fundus photograph of a seven-year-old girl with congenital rubella syndrome showing diffuse changes of salt and pepper pigmentary retinopathy throughout the fundus (white arrows). The patient also had subretinal hemorrhage with type 2 choroidal neovascular membrane (yellow arrow).^[205]^

### Alport Syndrome 

Alport syndrome is a hereditary genetic disorder due to mutations in the *COL4A3*, *COL4A4*, or *COL4A5* genes.^[163,164]^ These genes encode for collagen, specifically the alpha 3-5 chains of Type IV collagen, which is responsible for basal lamina formation.^[10,165,166]^ Multiple hereditary patterns are seen in Alport Syndrome, including autosomal recessive and dominant inheritance with the most common being X-linked dominant.^[167]^ It has an estimated prevalence of 1/5000 with 85% of patients presenting with the X-linked form.^[168]^ Alport syndrome is widely known for the clinical triad: hemorrhagic nephritis, SNHL, and ocular manifestations.^[169]^

Patients with X-linked disease typically present in childhood with asymptomatic microscopic hematuria, which is typically an incidental finding on urinalysis or is specifically screened for in a patient due to an affected family member. Patients may also present with gross hematuria which coincides after an upper respiratory infection.^[170]^

Several ocular manifestations occur in Alport Syndrome, the most common being dot-and-fleck retinopathy, anterior lenticonus, and posterior polymorphous corneal dystrophy.^[171]^ Other ocular defects include corneal erosion, microcornea, cataracts, posterior lenticonus, spontaneous lens rupture, spherophakia, among others.^[168,171]^ The majority of ocular symptoms stem from type IV collagen defects in membranes of the eye. For example, Descement's and Bowman's membrane are commonly affected, resulting in corneal dysfunction. The cornea fails to attach properly to the basement membrane, making abrasion and erosions likely. Patients present with progressive visual loss and pain due to corneal dystrophy and erosions, respectively. Patients may also present with astigmatism and decreased visual acuity. The manifestation of anterior lenticonus can present as lenticular myopia and is seen on direct ophthalmoscopy or slit-lamp as an “oil droplet sign”.^[172]^ Imaging via electron microscopy of anterior lenticonus is significant for multiple linear and irregular zones of dehiscence.^[173]^ Dot-and-fleck retinopathy is seen on fundoscopy ranges from a few scattered yellow or white dots and flecks to several densely accumulated dots and flecks in the perimacular region.^[174]^ A dull macular reflex, known as “lozenge”, can also present and is commonly associated with onset and progression of renal failure.^[174]^

Patients with Alport Syndrome typically experience features of SNHL during late childhood. Bilateral SNHL is common in patients with both the X-linked and autosomal recessive forms. The pathophysiology of this is also thought to be due to the defect in type IV collagen affecting inner ear structures, specifically the Organ of Corti within the cochlea.^[167]^ The progression of hearing loss begins with high frequency sounds and the rate of hearing loss also coincides with renal failure.

Early diagnosis of Alport Syndrome can be done via urinalysis (UA). Patients with multiple inheritance patterns have microhematuria, which can effectively be screened for by UA. Microalbuminuria and/or microproteinuria in Alport Syndrome can also be detected in UA.^[10,165,175]^ A significant family history of Alport Syndrome and hematuria can cause high suspicion for this disease process. Audiometry is a key diagnostic test that can be used to monitor changes in hearing. Confirmative diagnosis can be made via genetic testing or skin or kidney biopsy. Patients who have other family members with known genetic mutations can be tested for targeted mutations. Characteristic finding on kidney biopsy in patients with Alport Syndrome is splitting of the glomerular basement membrane on electron microscopy; immunofluorescence will show negative or nonspecific immunoreactant deposition to collagen type IV, which can provide both diagnostic and prognostic information.^[10]^ Skin biopsy findings can reveal absence of the alpha-5 chain of Type IV collagen via inability of monoclonal antibody to bind against the protein.^[10,165,176]^

Management of patients with Alport Syndrome consists of routine monitoring of renal function through serum electrolytes, GFR, and UA protein and red blood cell measurements.^[165]^ Early diagnosis is critical in monitoring progression of renal disease and allows for early intervention. Patients are usually started on an angiotensin II antagonist, such as lisinopril.^[175]^ Progressive renal failure can result in dialysis treatment or kidney transplantation.^[167]^ Patients should continue to follow-up with an ophthalmology specialist to progression of ocular defects. Those with corneal erosions and abrasions can be provided with topical solutions to prevent irritation of the cornea; however, persistent and severe corneal pathology may result in the need for corneal transplantation.^[7]^ Patients that present with anterior lenticonus may require lens replacement.^[7,171]^ Corrective lenses may also be provided in cases of decreased visual acuity due to myopia.^[7,171]^ Dot-and-fleck retinopathy has not been associated with any significant changes in visual acuity; thus, no treatment is necessary.^[174]^ Hearing aids and cochlear implants are good options for patients with SNHL.^[7]^

### Waardenburg Syndrome 

Waardenburg Syndrome (WS) is a hereditary group disorders that are characterized by pigmentary abnormalities of the hair, skin, and eyes with some degree of hearing loss.^[177,178,179,180]^ The prevalence of WS is 1/42,000 globally and approximately 2–5% of the congenitally deaf population.^[181,182]^ There are no differences in the prevalence with gender or race. There are four main subtypes of WS, the most common being type I and type II. A mutation in the WS gene occurs in transcription factors *PAX3* or *MITF*, which are responsible for the pathogenesis of WS Type I and WS Type II, respectively.^[181]^ Type III and IV WS are rare subtypes. Type III WS, also known as Klein–Waardenburg Syndrome, presents similarly type I with abnormalities in upper limbs.^[182]^ Type IV WS, known as Waardenburg–Shah Syndrome, occurs due to a mutation endothelin-3 receptor or SOX10 transcription factor and is associated with Hirschbrung's Disease.^[181,182]^ Type I, II, and III are inherited in an autosomal dominant pattern. Type IV is inherited in an autosomal recessive pattern.^[183]^

The leading theory of pathophysiology is thought to be due from an abnormal distribution of melanocytes during embryogenesis.^[181]^ This leads to patchy areas of depigmentation. Histopathology shows absence of melanocytes in hypopigmented areas.^[181]^ The depigmentation of hair and skin is typically seen in a piebald-like distribution.^[184]^ Noncutaneous features of WS include broad nasal root, abnormal pigmentation of the iris, and dystopic canthorum. WS type II can be distinguished from WS Type II via the absence of dystopia canthorum.

Ocular manifestations of WS may include dystopia canthorum, iris heterochromia, synophrys, ptosis, hypertelorism, strabismus, choroidal hypopigmentation, among others.^[185]^ The characteristic ocular presentation of WS is iris hypopigmentation, which can present as complete heterochromia, in which the iris of both eyes are different colors as well as partial heterochromia, in which only segments of the iris appear a different color.^[185]^ Stunted bright blue iris is also characteristic of WS.^[185,186]^ Dystopia canthorum presents as an abnormal distance between pupil and lateral canthi due to lateral displacement of the medial corner of the eyes.

Synophrys is characterized by a medial flaring of the hair of the inner portion of the eyebrows. Ptosis is a less commonly associated manifestation in WS.

The abnormalities of the Organ of Corti that lead to hearing loss in patients with WS more commonly presents bilaterally but may occur unilaterally. Patients with Type I and II WS are more commonly associated with SNHL. Histopathology of WS patients' ears is significant for atrophy of spinal ganglion and decreased nerve fibers in the absence of the Organ of Corti, which decreases sound vibrations to the brain.^[181]^

Waardenburg is a clinical diagnosis; however, patients are evaluated based on meeting major and minor criteria. There are five major and five minor criteria. Patients must meet two major criteria or one major criterion plus two minor criteria. Major criteria include white forelock, SNHL, iris pigmentation abnormality, dystopia canthorum, and an affected first degree relative. Minor criteria include skin hypopigmentation, synophrys, broad nasal root, hypoplastic nasal alae, and premature graying of the hair.^[187,188]^ Diagnosis can be confirmed with cytogenetic testing.

Management is primarily focused on symptomatology and genetic counseling. Coordinated treatment and management should involve dermatologist, ophthalmology specialists, hearing specialists, orthopedists, and gastroenterologists. Early diagnosis in children can improve developmental outcomes. Patients should be referred to an audiologist and a speech-language pathologist to prevent any delays in development. Hearing aids can be beneficial for patients with mild hearing loss; however, patients with low levels of hearing and language may benefit from cochlear implantation.^[189]^ Patients with skin and iris pigmentation should be advised to take preventative measures when exposed to sunlight, such as wearing sunscreen and sunglasses to prevent photosensitivity and risk for skin cancer. Patients with Type IV WS and Hirschbrung's disease may require surgical removal of the affected intestinal region.

##  STRENGTHS AND LIMITATIONS

The information presented in this review is largely heterogenous given the articles included, which vary in both context and design. The articles included range from case series, case reports, literature and systematic reviews, and clinical trials. This provides a strength given the rarity of each of these syndromes and the extensive compilation of their descriptions into this summary article. Despite this, the rarity of each of the syndromes may also pose a limitation in how patients are diagnosed and treated given the differences in population characteristics, geographic sites, and quality of publications that may have confounded our interpretation of the data.

##  SUMMARY

There are multiple syndromes that present with both ocular and SNHL findings. Patients that present with early symptoms of ocular or sensorineural hearing changes should not only be further assessed for the alternate but also assessed for any syndromic causes of their symptoms. Ophthalmologic and otolaryngologic consultation is pivotal in children to determine any specific deficits while also making sure patients are able to get timely interventions. The absence of eye-sight and/or hearing can have a dramatic impact on the sensory input a child receives. Thus, maximizing interventions, whether it is treatment and/or surgery, at an early age are pivotal to the development of the child.

##  Financial Support and Sponsorship

None.

##  Conflicts of Interest

The authors declare no potential conflicts of interest for this article's research, authorship, and/or publication.
